# Repeated recurrence of bilateral gigantomastia after subcutaneous mastectomy caused by tumoral pseudoangiomatous stromal hyperplasia: a case report and review of literature

**DOI:** 10.1259/bjrcr.20220074

**Published:** 2022-10-20

**Authors:** Manisa Sornlertlumvanich, Patteera Rohitopakarn, Srila Samphao, Varanatjaa Pradaranon, Warunee Kaewpiboon, Noppadol Kietsiriroje, Siriorn Danglaoun

**Affiliations:** 1 Department of Radiology, Faculty of Medicine, Prince of Songkla University, Songkhla, Thailand; 2 Department of Surgery, Faculty of Medicine, Prince of Songkla University, Songkhla, Thailand; 3 Endocrinology and Metabolism Unit, Department of Internal medicine, Faculty of Medicine, Prince of Songkla University, Songkhla, Thailand; 4 Department of Anatomical Pathology, Lampang hospital, Lampang, Thailand

## Abstract

Pseudoangiomatous stromal hyperplasia (PASH) is an uncommon benign proliferative mesenchymal lesion of the breast with a hormonal-sensitive nature. Various manifestations of PASH, ranging from an incidental microscopic finding in a tissue biopsy to a large palpable mass or bilateral gigantomastia, have been described. For tumoral PASH, surgical excision is indicated for a growing and symptomatic mass with a small chance of recurrence. A recurrence of bilateral gigantomastia after surgical excision or reduction mammoplasty is not common but has been occasionally reported, leading to further mastectomy. Repeated recurrence of bilateral gigantomastia is extremely rare. Herein, we report a case of a 13-year-old girl who presented with the third recurrence of bilateral gigantomastia caused by tumoral PASH, after undergoing bilateral reduction mammoplasty, and later subcutaneous mastectomy. Precocious puberty occurred early in this child at the age of 9 years, which may have been a factor unmasking PASH at this young age. The incomplete removal of the PASH could also have been a recurrence risk in our case as extended masses underneath the pectoralis muscle were later identified on the MRI study. This highlights the advantage of preoperative imaging in cases with a very large tumoral PASH in order to maximize the chance of complete tumor removal.

## Introduction

Macromastia or gigantomastia is a rare benign condition of excessive breast enlargement that has various definitions. Mostly, it is considered when there is more than 1.5–2.5 kg of excessive breast tissue,^1^ or the tissue contributes more than 3% of body weight.^2^ This condition has mainly been reported in pubertal or premenopausal females and frequently leads to physical and/or psychological disturbances.^3,4^ The etiologies of gigantomastia according to the proposed subclassification by Dancey et al. in 2008 are 1) idiopathic spontaneous breast growth, 2) endogenous hormone stimulation, and 3) drug-induced gigantomastia. However, other conditions such as benign tumors like fibroadenoma, phyllodes tumor and papillomatosis, or even a more serious condition like breast carcinoma, are also included in the differential diagnosis, thus they need to be ruled out.^4–6^ Among the benign conditions, there is an uncommon condition named pseudoangiomatous stromal hyperplasia (PASH) which has various manifestations ranging from a focal microscopic change on biopsy, to a focal mass/multiple masses, or a rare diffuse form.^1,7–10^ Despite the benignity of PASH, surgical excision is usually required when the lesion is symptomatic or imaging-pathological discordant suggesting a more serious condition such as cancer.^11–13^ For a PASH that manifests as bilateral gigantomastia, mastectomy is recommended to completely remove the lesions^7,8,14–18^ to reduce the chance of recurrence.^12^ Although a small chance of recurrence after subcutaneous mastectomy still exists, multiple recurrences of PASH are extremely rare (Table 1). To our knowledge, only two cases with multiple recurrences of PASH have been reported.^14,19^

**Table 1.** Case reports of recurrent bilateral gigantomastia caused by PASH(^12,14–21^)

Author(s)	Age of presentation	Presentation	Disease progression	Treatment	Final outcome
Kim et al.,2018	33	Excessive breast enlargement 2 years after pregnancy No hormonal profile	- Continuous enlargement of both breasts without palpable discrete mass(es)	- Reduction mammoplasty	- No recurrence after bilateral mastectomies
			- Recurrent gigantomastia after 4 years	- Bilateral mastectomies	
Singh et al.,2007	12	Asymmetrical bilateral breast enlargement for 4 months following start of menarche No hormonal profile	- More prominent left breast enlargement	- Left breast excision	- No recurrence after bilateral mastectomies
			- Continuous enlargement of the right breast	- Right subcutaneous mastectomy (85% removal)	
			- Re-enlargement of both breasts after surgery	- Left breast reduction to correct the asymmetry	
			- Second re-enlargement of both breasts after surgery	- Bilateral mastectomies	
Xu et al.,2020	44	Enlargement of both breasts following pregnancy No hormonal profile	- Rapid enlargement of both breasts and right axillary breast tissue following pregnancy	- Reduction mammoplasty followed by right axillary breast excision	- No recurrence after bilateral mastectomies
			- Rapidly re-enlarged breasts within 8 months after the initial surgery, with more pronounced left axillary breast tissue	- Bilateral mastectomies and left axillary breast tissue resection	
Vasconceloset al., 2015	45	Palpable right breast mass Pre-menopause No history of taking relevant medications	- Right breast mass, seen on ultrasound and MRI	- Excision	- No recurrence after bilateral mastectomies
			- Palpable masses in both breasts in 1 year	- Bilateral tumor excisions	
			- Bilateral breast masses 2 years later resulting in macromastia	- Bilateral mastectomies with immediate breast reconstruction with expanders	
Lee et al.,2016	41	- Painful and swollen breasts for 2 months	- Excessively enlarged breasts without discrete masses	- Bilateral reduction mammoplasty	- Lost to follow up
			- Increased breast volumes to the preoperative size within 6 months	- Refused additional surgical treatment	
Pruthi et al.,2001	39	Progressive bilateral breast enlargement with tenderness and erythema No history of oral contraceptive use	- Bilateral asymmetrical enlargement with a palpable mass in the right axilla and left breast	- A trial of tamoxifen for 1 year with improvement of erythema, pain and breast size	- Decreased breast pain and engorgement with continuation of daily Tamoxifen
			- Recurrence of bilateral breast pain and engorgement as well as palpable masses in both breasts after drug discontinuation	Reduced-dose tamoxifen for 6 months due to side-effects of the drug, then changing to raloxifene for 1 year with disappearance of the mass in the right breast Changing to tamoxifen, after development of side-effects of raloxifen,	
Roy et al.,2015	40	- Bilateral cyclical breast swelling	- Bilateral breast enlargement and presence of mobile nodules	- tamoxifen 10 mg for 4 months	- Unremarkable postoperative course
			- Continuous enlargement of breasts despite medical management	- Bilateral breast reduction with free nipple graft (pathological results: PASH and giant fibroadenoma)	
Bourke et al.,2015	46	Bilateral asymmetrical breast enlargement for 6 weeks Using contraceptive coil for 6 months	- Markedly edematous breasts without discrete masses	- Bilateral mammoplasty with image-guided localization of the suspected lesions	- No recurrence after bilateral mastectomies
			- Enlargement of the breasts 3 years later	- Bilateral mastectomies with immediate reconstruction	
Smaila et al.,2018	34	Progressive painless bilateral enlargement of breasts No hormonal profile	- Bilateral breast enlargement for 1 year	Excision of masses in both breasts No pathological result	- No recurrence during a 6 month follow-up
			Accelerated enlargement a few weeks after surgery No palpable mass	- Bilateral mastectomies	

Herein we present a case of PASH in a 13-year-old girl who had three recurrences of bilateral gigantomastia after two surgeries.

## Case presentation

**Figure 1.**
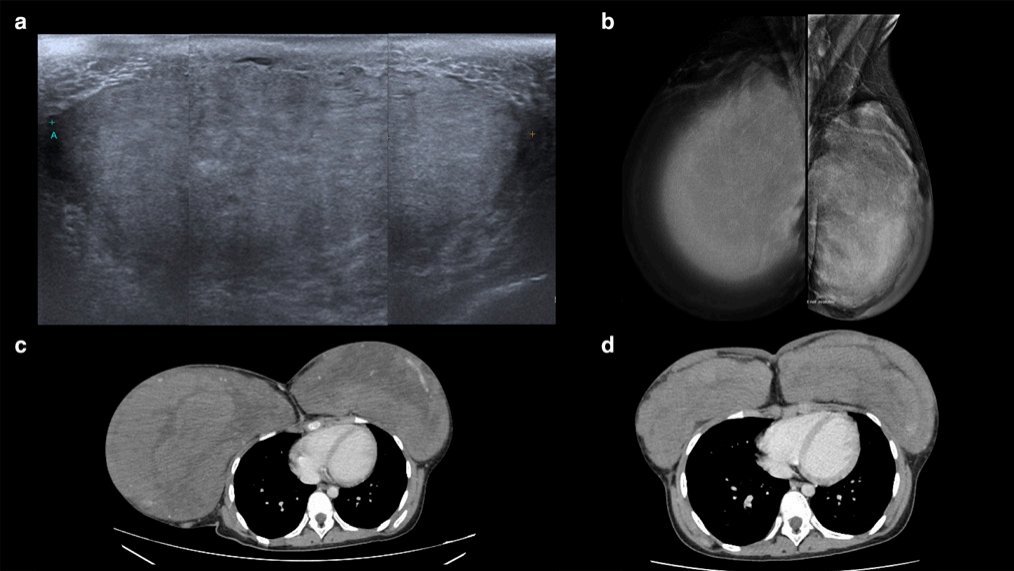
(**A-D**) An 11-year-old girl with bilateral breast enlargement. (**A**) Breast ultrasound shows a large well-defined heterogeneous mass in the right breast, measuring 15 cm. (**B**) On mammogram, MLO view, the breasts are extremely dense and diffusely enlarged. (**C**) Axial preoperative chest CT shows a large heterogeneously enhanced mass occupying each entire breast, more prominent on the right. (**D**) These masses re-enlarge 4 months following reduction mammoplasty.

A 13-year-old premenarchal girl presented at our hospital with recurrent excessive bilateral breast enlargement 6 months after a second surgery at another hospital 10 months previously. Her history of abnormal breast enlargement had started when she was only 9 years old and not taking any relevant hormonal therapy. The physical examination at that time found Tanner Stage 4, specifically bilateral breast enlargement with firm to hard consistency, which was discordant with her age. At first, her primary physician assessed her condition as rapid, progressive breast growth from precocious puberty. Initial investigations revealed an abnormally increased LH level (1.82 mIU/mL, normal reference≤0.69 mIU/mL for her age). Other hormones were also measured and showed a normal FSH level (4.71 mIU/mL, reference range 0.72–5.33 mIU/mL) and slightly high estradiol level (26.6 pg ml^−1^, reference range<25 pg ml^−1^). She was given a gonadotropin-releasing hormone (GnRH) analog to inhibit the pubertal process for a year, resulting in breast size reduction and a 12-cm height increase. Six months after the treatment discontinuation, at age 11 years, her breasts resumed their rapid, asymmetrical growth. A repeated hormonal profile showed all results within a normal range for her age including LH (2.46 mIU/mL, reference range≤4.38 mIU/mL), FSH (3.9 mIU/mL, reference range 0.87–9.16 mIU/mL) and estradiol (14 pg ml^−1^, reference range<25 pg ml^−1^). The asymmetrical breast enlargement led her physician to investigate for tumors. Breast ultrasound revealed a large mass in each breast, up to 15 cm in maximal diameter (Figure 1A), which appeared as bilateral extremely dense breast tissue on mammogram. Preoperative chest CT was also performed, showing a large heterogeneously enhanced mass occupying each entire breast, more prominent on the right (Figure 1B–C). The pathology from incisional biopsies of the masses in both breasts suggested PASH, after which medial pedicle reduction mammoplasty was performed. However, her breasts again re-enlarged within 4 months, (Figure 1D), and this time bilateral subcutaneous mastectomies with preserved alveolar complexes were performed. Totals of 4600 gm and 1,700 gm of tissue were removed from the right and left breasts, respectively.

**Figure 2.**
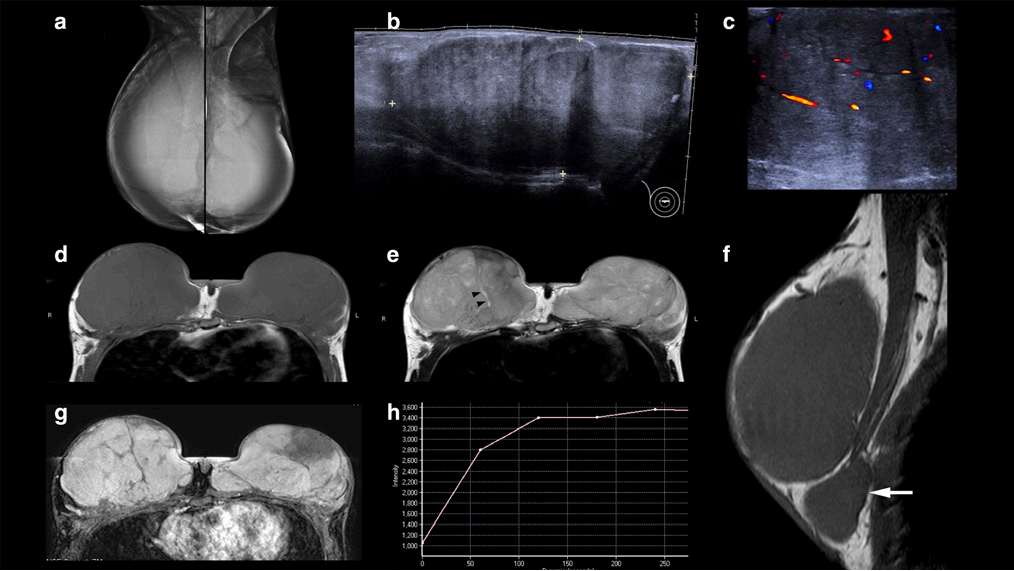
(**A-H**) A 13-year-old girl with bilateral PASH. (**A**)The mammogram reveals extremely dense breasts, due to a large equal-density mass in each breast. (**B**) Panoramic view ultrasonography of the right breast shows a large, oval, circumscribed, heterogeneous hypoechoic mass, measuring 13 cm in the longest diameter. (**C**) Doppler ultrasonography shows increased internal vascularity of the mass. On MRI, there is a large oval circumscribed iso-intensity mass occupying each entire breast on (**D**) axial *T_1_*-weighted and (**E**) *T_2_*-weighted images with internal hypersignal slit-like spaces (arrowheads). (**F**) A sagittal *T_1_*-weighted image of the right breast shows a smaller mass (arrow) underneath the pectoralis muscle. The other breast had a similar mass (not shown) (**G**) Axial contrast-enhanced *T_1_*-weighted image at 2 min shows fair homogeneous enhancement of the masses. (**H**) A time-intensity dynamic contrast enhancement curve of the enhancing mass shows a Type 1 delayed persistent pattern.

Six months after the second surgery, she was referred to our hospital because of the third recurrence of bilateral gigantomastia. Both mammogram and ultrasonography were performed and showed a large, oval, circumscribed mass in each breast. (Figure 2A–C) The MRI study of both breasts revealed a few large, oval, circumscribed, variable-sized masses which exhibited iso-intensity on both *T_1_-* and *T_2_*-weighted images. On the *T_2_*-weighted images, thin hypersignal slit-like spaces were found within both masses. A Type 1 enhancement pattern on post-gadolinium injection images was also seen in both breasts. Some of these masses extended beneath the pectoralis muscle. (Figure 2D–H)

**Figure 3.**
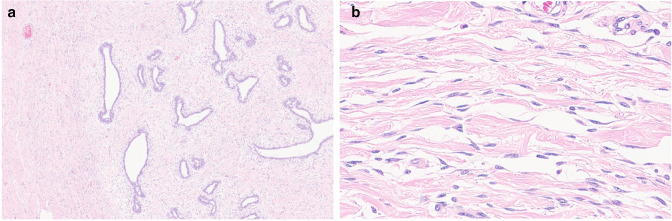
(**A-B**) Microscopic examination of the masses from Figure 2. (**A**) On low-power view (hematoxylin & eosin stain, (**x10**), a grossly well-demarcated nodular PASH with internal ducts and fibrous stroma can be seen. (**B**) On high-power view (hematoxylin & eosin stain, x100), multiple slit-like spaces or pseudovascular spaces can be seen within hyperplastic stroma. These pseudovascular spaces are lined by stromal myofibroblasts without nuclear atypia.

Since the patient was long suffering from her extremely large breasts, with her parents’ consent, she agreed to undergo bilateral simple mastectomies to completely remove the tumors, following which the resection margins were free from tumor. The pathological report confirmed the diagnosis of tumoral PASH in both breasts (Figure 3A–B). No recurrence of the breast enlargement had occurred by the time this case report was written over a year following the final surgery, which was accounted as at least 12-month recurrence-free. A timeline of the presentations and overall management is shown in Table 2.

**Table 2.** summary of the study patient’s timeline

Visit	Age	Presentation	Hormonal tests	Imaging studies	Management	Outcome
1	nine y 9 m	Excessive breast enlargement No menarche	LH 1.82 mIU/mL (≤ 0.69 mIU/mL) FSH 4.71 mIU/mL (0.72–5.33 mIU/mL) Estradiol 26.6 pg ml^−1^ (<25 pg ml^−1^)	- None	- GnRH analogue injections for 1 year	- Temporary decreases in size of breasts during the treatment
2	eleven y 11 m	- Rapidly and asymmetrically enlarged breasts within 6 months	LH 2.46 mIU/mL (≤ 4.38 mIU/mL) FSH 3.9 mIU/mL (0.87–9.16 mIU/mL) Estradiol 14 pg ml^−1^ (<25 pg ml^−1^)	US: a few large masses in both breasts Mammogram: extremely dense and diffusely enlarged breasts CT: heterogeneously enhanced masses in both breasts	- Incisional biopsy followed by medial pedicle reduction mammoplasty	- Pathological result: PASH
3	12 y 6 m	- Re-enlarged breasts within 4 months after mammoplasty	- None	- CT: re-enlargement of the heterogeneously enhanced masses in both breasts with skin thickening from post-surgery	- Bilateral subcutaneous mastectomies with preserved alveolar complexes	- Pathological result: PASH
4	13 y	- third recurrence of bilateral gigantomastia 6 months after surgery	- None	US: oval, circumscribed, hypoechoic mass in each breast with increased internal vascularity Mammogram: extremely dense breasts MRI: a large oval circumscribed homogenous enhanced mass occupying each entire breast with high-T2 slit-like spaces. A few smaller masses underneath the pectoralis muscle on both sides	- Bilateral simple mastectomies	Pathological result: PASH No recurrence during the 1 year follow-up

CT, computed tomography; FSH, follicle-stimulating hormone; GnRH, gonadotropin-releasing hormone; LH, luteinizing hormone; MRI, magnetic resonance imaging; PASH, pseudoangiomatous stromal hyperplasia; US, ultrasound.

## Discussion

PASH is an uncommon benign proliferative mesenchymal lesion without a clearly understood pathogenesis.^22,23^ It was first described by Vuitch et al. in 1986.^24^ The presentations of PASH are non-specific, ranging from incidental microscopic findings through a diffuse process without obvious masses in imaging to clinically palpable masses.^23^ It is incidentally found from tissue biopsies of other lesions in up to 23% of cases.^25^ Occasionally, it can manifest in a mass form which is usually a unilateral, discrete, slow-growing mass.^22,26^ Rarely, PASH can present with multiple masses causing asymmetrical, bilateral breast enlargement, or diffuse bilateral enlargement without a discrete mass.^7,8,10,22^

Histopathological confirmation is always required for the diagnosis due to its non-specificity. The histology of PASH is proliferation of stroma with fibroblasts and myofibroblasts, as well as dilated or slit-like interanastomosing spaces lined by slender spindle cells. These spaces resemble vascular channels except for the absence of endothelial markers.^11,22–24^ Due to its pseudovascular channels, PASH is also known as a histological mimicker of low-grade angiosarcoma.^23^ The etiology of PASH could be an excessive response of myofibroblasts in breast tissues to hormone stimulation, especially progesterone. This hypothesis is supported by the frequently positive staining of progesterone and/or estrogen receptors in PASH, explaining why this disease has been mostly found in adolescents, premenopausal females and older females on hormonal therapy,^11,22,23^ as well as patients who have recently had a hormonal surge event such as recent menarche or pregnancy.^12,15,19^ Despite the fact that the PASH-caused gigantomastia in our case arose during the prepubertal period, the dramatic increase in the initial hormonal profiles of this patient, pointing toward precocious puberty, could stimulate the rapid growth of the tumors at this young age, and conceal the disease leading to a diagnostic challenge.

Imaging manifestations of PASH are also various and non-specific regardless of imaging modality. Generally speaking, the role of imaging studies is to evaluate the nature of a lesion and to exclude other malignant conditions rather than making a definite diagnosis of PASH. Yet there are common features of tumoral PASH reported for different imaging modalities. On mammography, the tumoral PASH usually presents as a round or oval mass with a circumscribed or partly circumscribed margin. This manifestation, however, can be found in more common diseases such as fibroadenoma.^22,23,27^ On the other hand, calcification is rarely reported in PASH, hence it may be used as a negative predictor for PASH. Global or focal asymmetrical breast enlargement without a discrete mass is less frequent and can be found in patients with diffuse PASH.^14,28^ On ultrasound, PASH is commonly seen as a circumscribed hypoechoic mass. Its internal echogenicity may be heterogeneous with various posterior features, mimicking fibroadenoma.^22,23,27^ Less commonly, it can be an ill-defined or hyper-echogenic mass. Internal cystic components or vascular channels, when they are found, may help in differentiating PASH from fibroadenoma.^13^ On MRI, these masses usually show low signal intensity on T1w images and various intensity signals on T2w images. When a hypersignal reticular pattern on a T2w image, indicating slit-like spaces within the lesion, is found, a diagnosis of PASH is favored. The pattern of enhancement can be either a mass or a non-mass enhancement. The kinetic curve of the persistent enhancement pattern is often reported, suggesting its benignity.^23^

A differential diagnosis for PASH includes the following diseases. First is fibroadenoma - a very common disease that can manifest with abnormal breast enlargement in young females. Given the similarity in both clinical and imaging features between PASH and fibroadenoma,^7,22,23^ particularly the juvenile type which can present with multiple bilateral masses as in our case, fibroadenoma, therefore, is always included in the differential diagnosis for tumoral PASH.^6^ Second, phyllodes tumor should also be included in the list, especially when the presentation is a rapidly growing mass, despite its infrequent occurrence in adolescents.^14^ Third, when the patient presents with rapid bilateral breast enlargement without discrete masses, juvenile gigantomastia (also known as virginal breast hypertrophy) is another condition that we should be aware of and consider in a differential, since several treatment options are available apart from mastectomy, especially hormonal therapy for the juvenile type.^6^ It should be emphasized that these various diseases cannot be differentiated by only single clinical or imaging features, and thus tissue histopathology is required for definite diagnosis.

The current treatment for PASH ranges from simple observation for incidental small lesions to surgical excision which is indicated when: 1) tumor size is larger than 2–3 cm in diameter, 2) the tumor is rapidly growing in nature, or 3) clinical presentation or imaging findings are suspicious for malignancy.^11–13,29^ Recurrence rates after excision have been reported ranging from 0 to 28.5%, leading to re-excision.^22,25,26^ Tumoral PASH is usually dormant or slow growing in nature, however, in uncommon tumoral PASH, the lesions can grow rapidly yet without malignant potential.^30,31^ The growth rates of PASH as assessed by various consecutive imaging studies such as breast ultrasonography or mammography have ranged from 0–71.4%,^26^ but it is very uncommon for PASH to rapidly grow causing bilateral gigantomastia.^9,10^

When gigantomastia develops, mastectomy is usually required to ensure complete PASH tissue removal^7,8,14,18^ and incomplete removal can be one of the risks for recurrence (Table 1). From our literature search, only 9 cases of PASH presenting with recurrent gigantomastia have been reported (Table 1).^12,14–21^ Among those, only two cases had repeated recurrences.^14,19^ Seven out of the 9 cases underwent one of the partial tissue removal techniques (mammoplasty, surgical excision or subcutaneous mastectomy) as an initial treatment before the tumor regrew.^12,14–19^ Six patients eventually had bilateral mastectomies resulting in complete remission.^12,14–17,19^ In our case, a few small masses extending underneath the pectoralis muscle were visualized in the preoperative MRI. This highlights the role of preoperative imaging, particularly MRI, in treatment planning to maximize the likelihood of complete tumor removal and minimize the chance of recurrence.

Medical treatment could have a role in symptom and tumor size control in some cases. Pruthi et al reported a case given tamoxifen as an alternative treatment for PASH with positive estrogen receptors, based on the hypothesis that PASH is sensitive to estrogen stimulation.^21^ They reported that the tamoxifen reduced the patient’s breast size within two months but with tumor recurrence after 1 year of drug discontinuation. On the other hand, Roy et al reported a case in which tamoxifen was ineffective for tumor size control, necessitating breast reduction surgery.^20^ Other hormonal treatments inhibiting GnRH secretion such as bromocriptine, medroxyprogesterone, and danazol have also been used for pre- and postoperative control in patients with juvenile gigantomastia, since the breast tissues are hormonal-sensitive.^3^ Such drugs can cause regression of hypertrophied tissue, slowing and stopping breast growth. However, the use of these drugs has not yet been studied in PASH. In our patient, the GnRH analogue was initially given to delay the precocious puberty, thus inhibiting breast growth and the hidden tumoral PASH. The GnRH analogue was able to suppress breast enlargement during the treatment course, but her breasts started to re-enlarge again after the drug was discontinued. This emphasizes the role of hormonal stimulation in PASH. Therefore, hormonal therapies inhibiting GnRH secretion might be another alternative hormonal treatment or an adjuvant treatment to surgical management for PASH in prepubertal girls. However, further research is required before any firm conclusions can be made.

## Conclusion

We report a case of tumoral PASH manifesting with a rare aggressive nature, as excessive and rapidly growing bilateral gigantomastia, in a prepubertal girl formerly with precocious puberty. Although tumoral PASH in general is dormant and unlikely to cause gigantomastia, hormonal axis activation during the precocious puberty may have stimulated the excessive tumor growth in our patient. That the use of a GnRH analogue was able to suppress the tumor growth supports the hormonal stimulation hypothesis of PASH. Triple recurrences occurring after mammoplasty and subcutaneous mastectomy suggests that complete tumor removal by simple mastectomy is a more favorable approach in cases of PASH-caused gigantomastia with diffuse disease or a very large mass, particularly in cases with aggressive disease. MRI may be a useful tool to evaluate the extent of and guide treatment planning to ensure complete tumor removal thus reducing the chance of recurrence.

## Learning points

- PASH is an uncommon benign condition that rarely presents as bilateral gigantomastia.
- Due to the hormonal sensitivity of PASH, hormone stimulation during precocity is also considered to be a factor that can unmask PASH in prepubertal girls.
- A definite diagnosis of PASH cannot be made by imaging findings alone. However, imaging studies, especially MRI, are useful in evaluating malignant features and tumor extension in order to provide proper treatment.
- Due to the risk of recurrence after incomplete surgical removal in cases of PASH-caused gigantomastia, mastectomy is often indicated, despite the general benignity of this disease.
